# Supplementary material for: A ribosomal protein S5 isoform is essential for oogenesis and interacts with distinct RNAs in Drosophila melanogaster
Source: Sci Rep. 2019 Sep 24;9:13779. doi: 10.1038/s41598-019-50357-z (PMC6760144; doi:10.1038/s41598-019-50357-z)
Supplement: Supplementary file 1 — Supplementary Figures 1–6 [file 41598_2019_50357_MOESM1_ESM.pdf]

Supplementary Information for:

A ribosomal protein S5 isoform is essential for oogenesis and interacts with distinct RNAs in *Drosophila melanogaster*

Jian Kong, Hong Han, Julie Bergalet, Louis Philip Benoit Bouvrette, Greco Hernández, Nam-Sung Moon, Hojatollah Vali, Éric Lécuyer, and Paul Lasko

Contents:

6 Supplementary Figures (S1 through S6)

Legends to Supplementary Figures S1 through S7

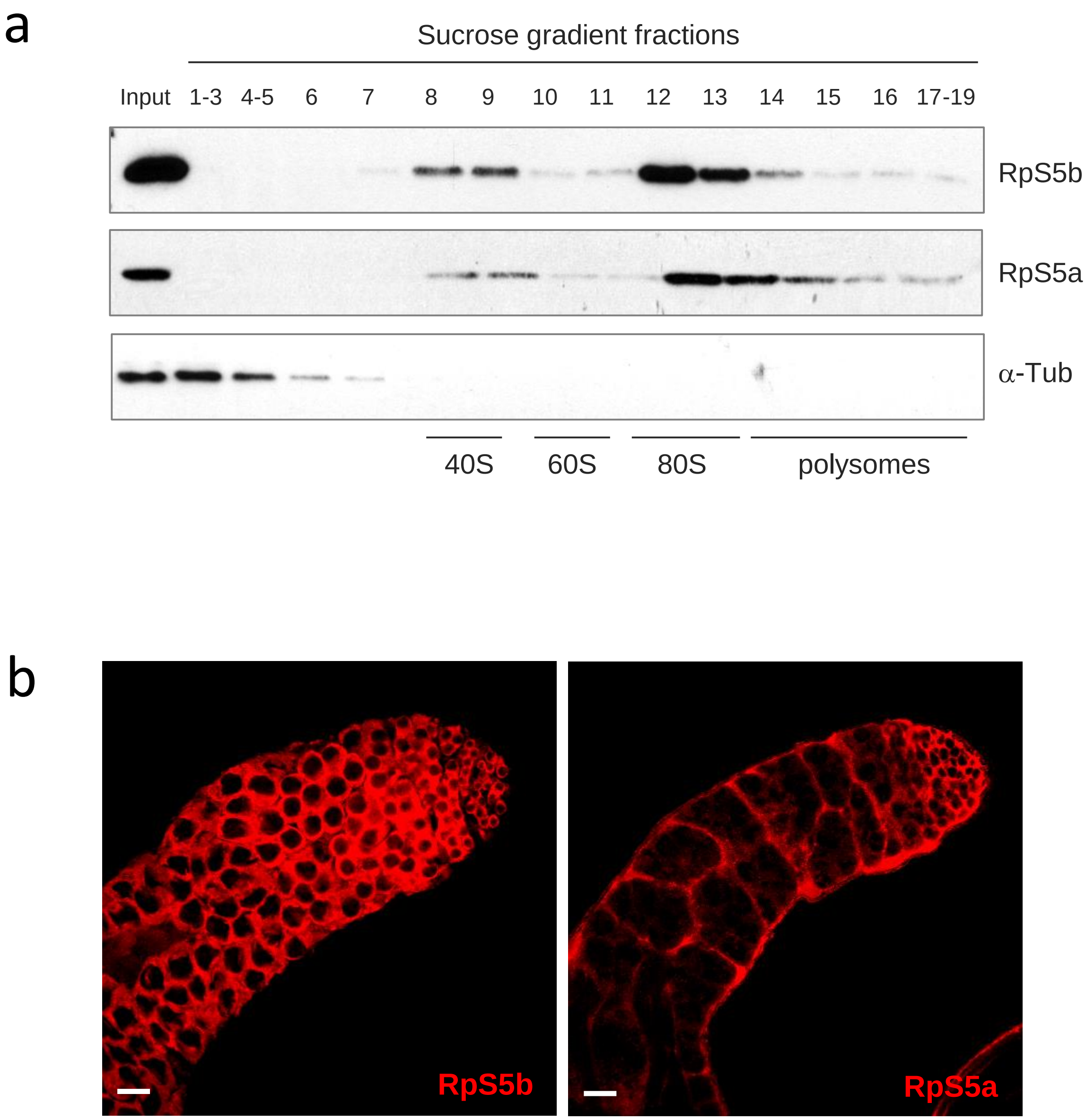
a
Sucrose gradient fractions
Input
1-3
4-5
6
7
8
9
10
11
12
13
14
15
16
17-19
RpS5b
RpS5a
α-Tub
40S
60S
80S
polysomes
b
RpS5b
RpS5a

Figure S1

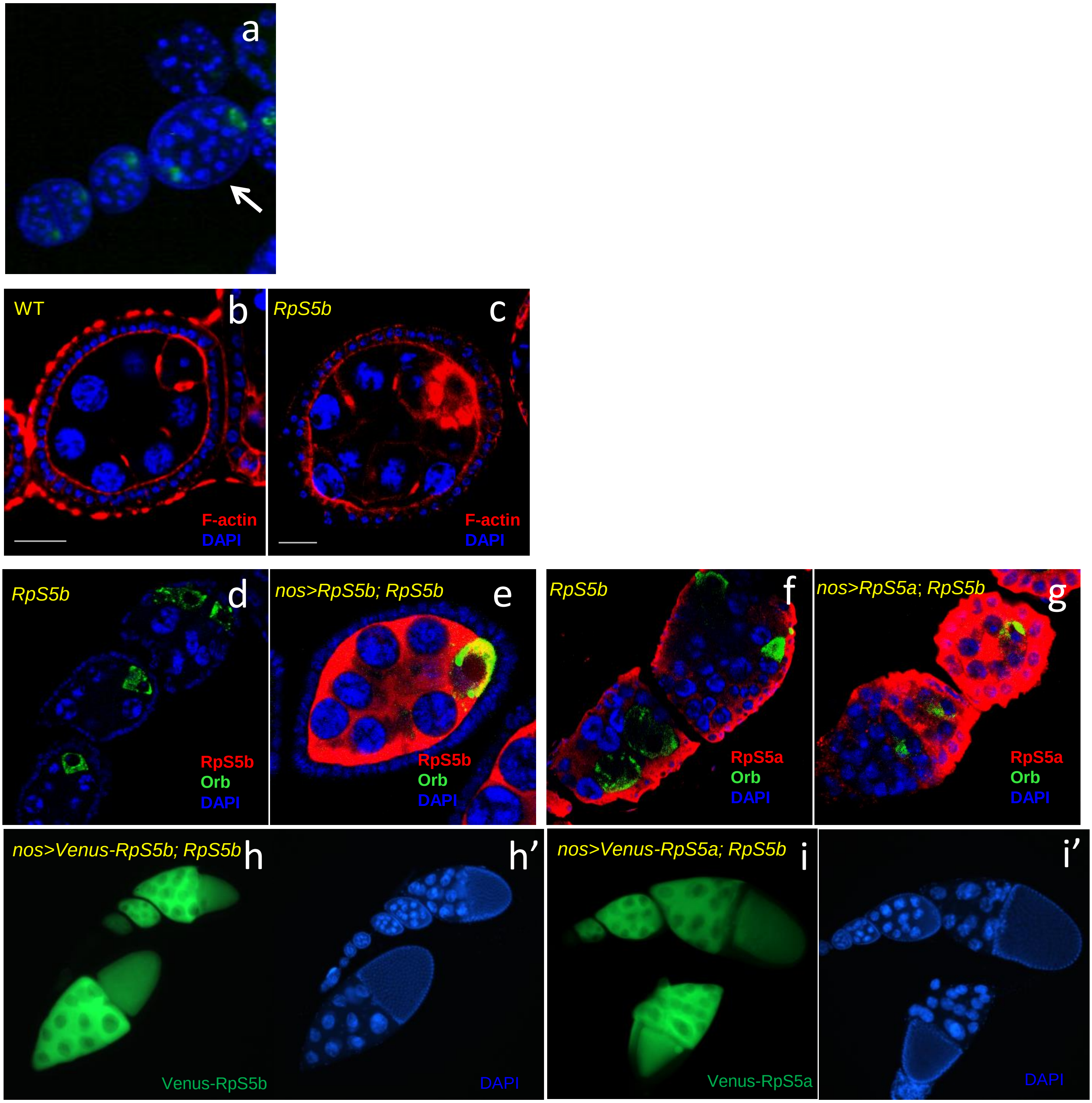
a
WT
b
F-actin
DAPI
RpS5b
c
F-actin
DAPI
RpS5b
d
RpS5b
Orb
DAPI
nos>RpS5b; RpS5b
e
RpS5b
Orb
DAPI
RpS5b
f
RpS5a
Orb
DAPI
nos>RpS5a; RpS5b
g
RpS5a
Orb
DAPI
nos>Venus-RpS5b; RpS5b
h
h'
Venus-RpS5b
DAPI
nos>Venus-RpS5a; RpS5b
i
i'
Venus-RpS5a
DAPI

Figure S2

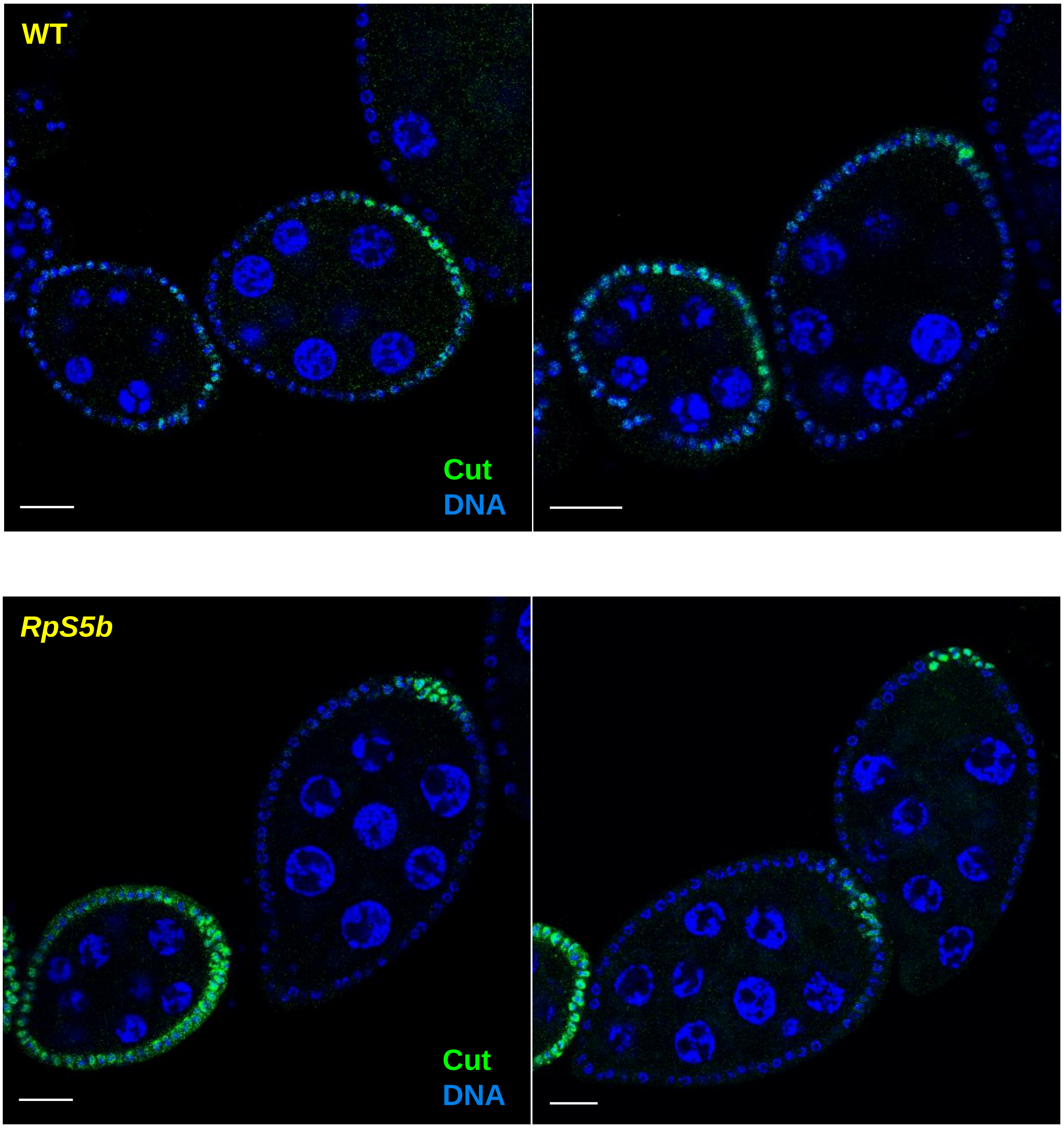

Figure S3

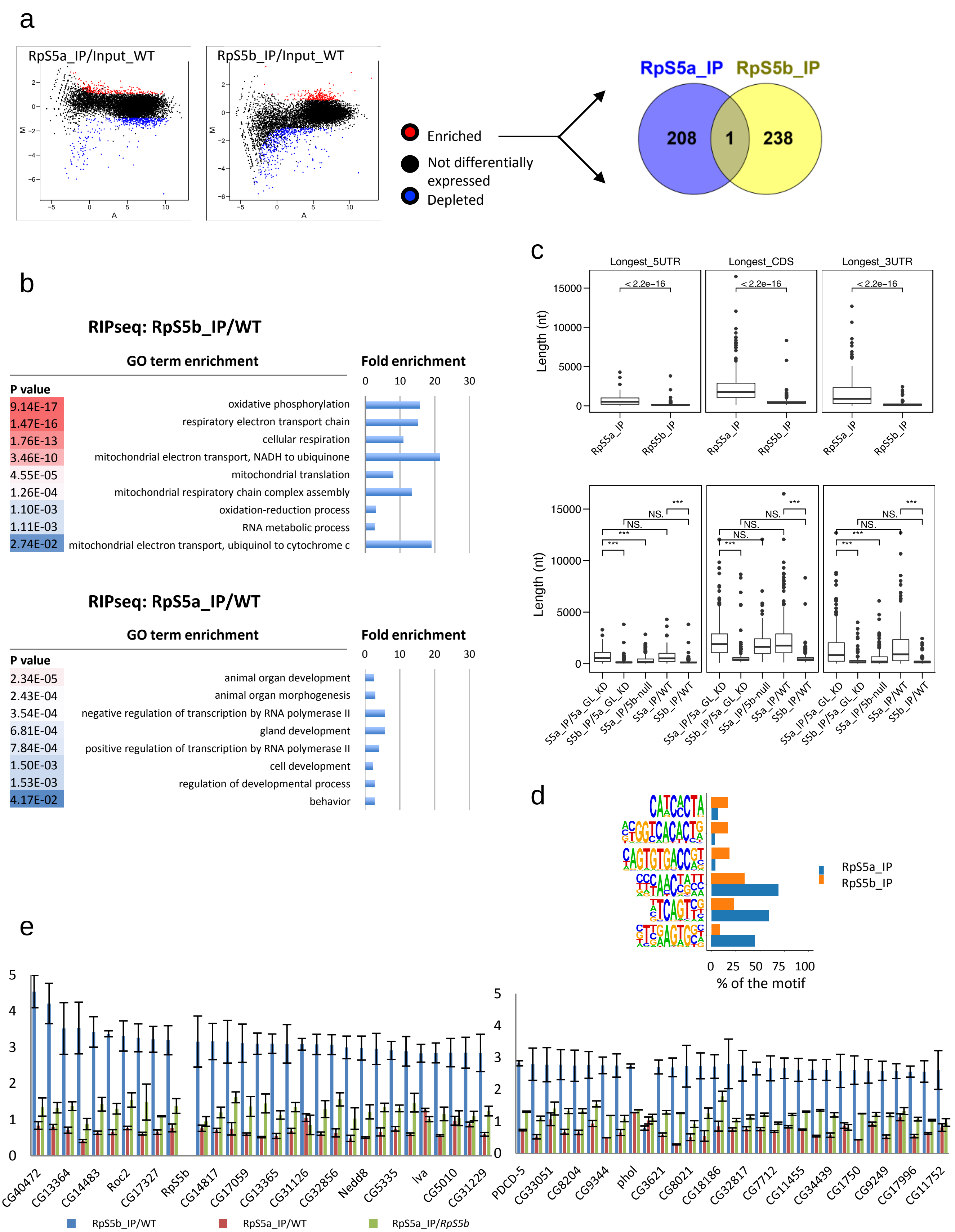

a
RpS5a_IP/Input_WT
RpS5b_IP/Input_WT
Enriched
Not differentially expressed
Depleted
RpS5a_IP
RpS5b_IP
208
1
238
b
RIPseq: RpS5b_IP/WT
GO term enrichment
Fold enrichment
P value
9.14E-17 oxidative phosphorylation
1.47E-16 respiratory electron transport chain
1.76E-13 cellular respiration
3.46E-10 mitochondrial electron transport, NADH to ubiquinone
4.55E-05 mitochondrial translation
1.26E-04 mitochondrial respiratory chain complex assembly
1.10E-03 oxidation-reduction process
1.11E-03 RNA metabolic process
2.74E-02 mitochondrial electron transport, ubiquinol to cytochrome c
RIPseq: RpS5a_IP/WT
GO term enrichment
Fold enrichment
P value
2.34E-05 animal organ development
2.43E-04 animal organ morphogenesis
3.54E-04 negative regulation of transcription by RNA polymerase II
6.81E-04 gland development
7.84E-04 positive regulation of transcription by RNA polymerase II
1.50E-03 cell development
1.53E-03 regulation of developmental process
4.17E-02 behavior
c
Longest_5UTR
Longest_CDS
Longest_3UTR
Length (nt)
< 2.2e-16
RpS5a_IP
RpS5b_IP
S5a_IP/5a_GL_KD
S5b_IP/5a_GL_KD
S5a_IP/5b-null
S5a_IP/WT
S5b_IP/WT
NS.
***
d
RpS5a_IP
RpS5b_IP
% of the motif
e
CG40472
CG13364
CG14483
Roc2
CG17327
RpS5b
CG14817
CG17059
CG13365
CG31126
CG32856
Nedd8
CG5335
Iva
CG5010
CG31229
PDCD-5
CG33051
CG8204
CG9344
phol
CG3621
CG8021
CG18186
CG32817
CG7712
CG11455
CG34439
CG1750
CG9249
CG17996
CG11752
RpS5b_IP/WT
RpS5a_IP/WT
RpS5a_IP/RpS5b

Figure S4

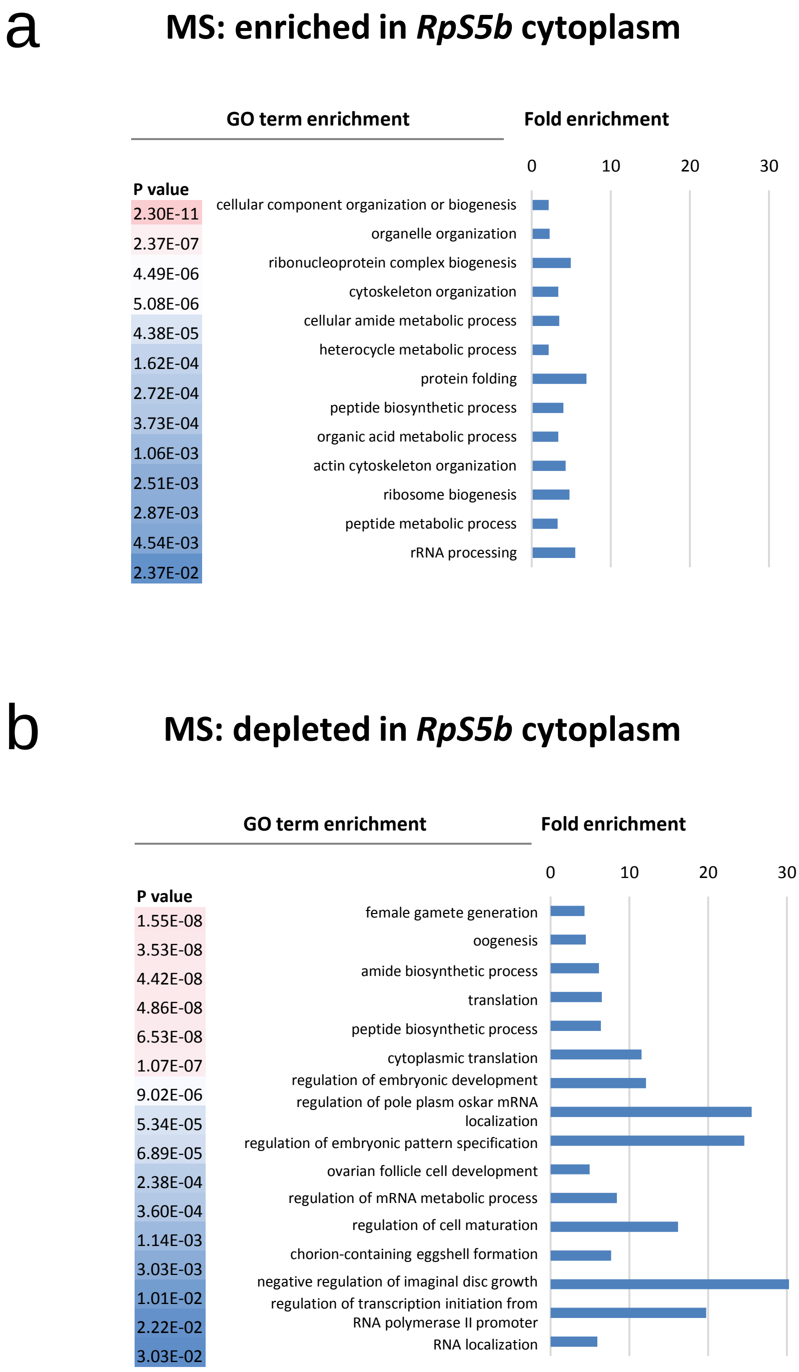

a
MS: enriched in RpS5b cytoplasm
GO term enrichment
Fold enrichment
0
10
20
30
P value
2.30E-11
cellular component organization or biogenesis
2.37E-07
organelle organization
4.49E-06
ribonucleoprotein complex biogenesis
5.08E-06
cytoskeleton organization
4.38E-05
cellular amide metabolic process
1.62E-04
heterocycle metabolic process
2.72E-04
protein folding
3.73E-04
peptide biosynthetic process
1.06E-03
organic acid metabolic process
2.51E-03
actin cytoskeleton organization
2.87E-03
ribosome biogenesis
4.54E-03
peptide metabolic process
2.37E-02
rRNA processing
b
MS: depleted in RpS5b cytoplasm
GO term enrichment
Fold enrichment
0
10
20
30
P value
1.55E-08
female gamete generation
3.53E-08
oogenesis
4.42E-08
amide biosynthetic process
4.86E-08
translation
6.53E-08
peptide biosynthetic process
1.07E-07
cytoplasmic translation
9.02E-06
regulation of embryonic development
5.34E-05
regulation of pole plasm oskar mRNA localization
6.89E-05
regulation of embryonic pattern specification
2.38E-04
ovarian follicle cell development
3.60E-04
regulation of mRNA metabolic process
1.14E-03
regulation of cell maturation
3.03E-03
chorion-containing eggshell formation
1.01E-02
negative regulation of imaginal disc growth
2.22E-02
regulation of transcription initiation from RNA polymerase II promoter
3.03E-02
RNA localization

Figure S5

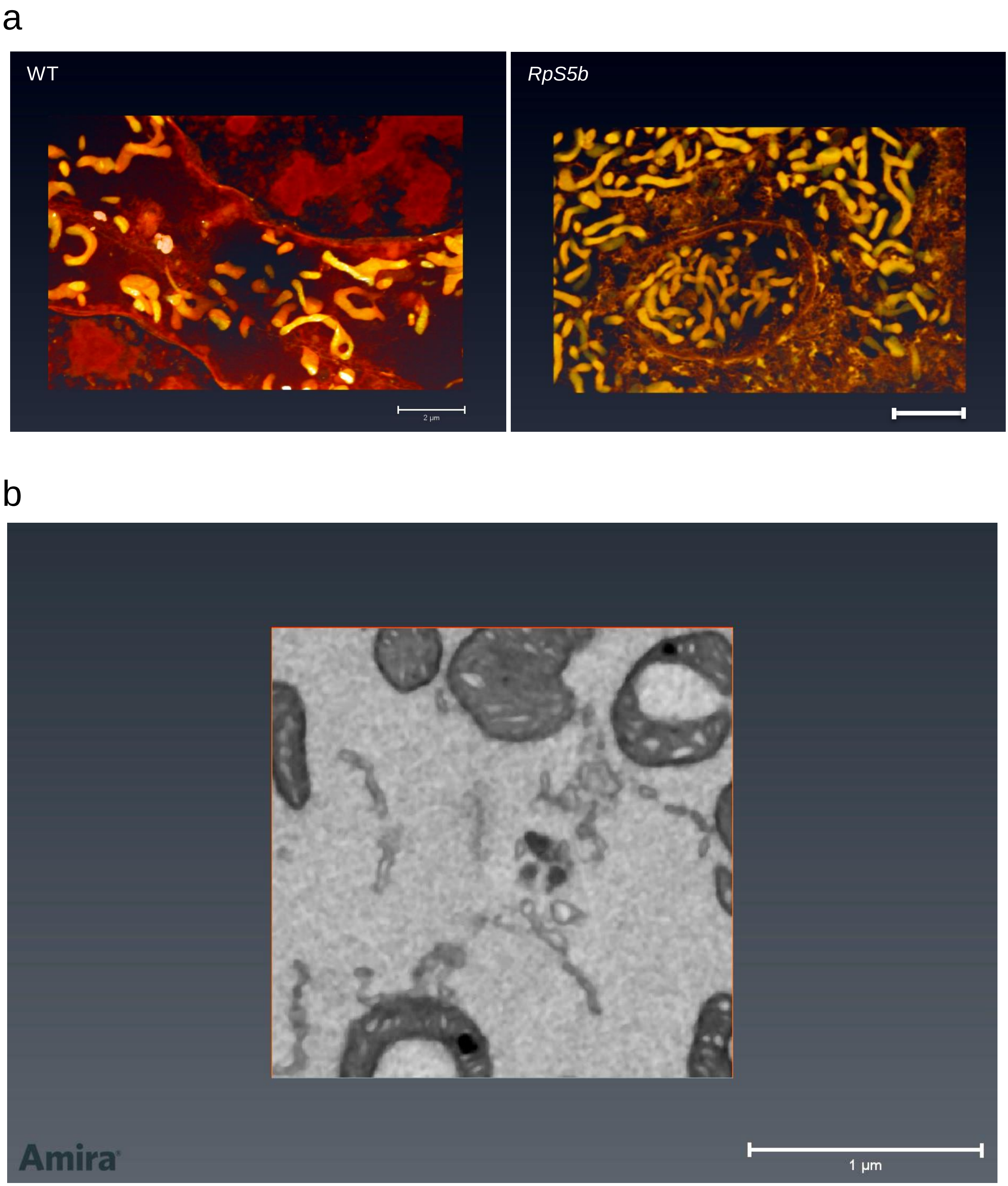

Figure S6

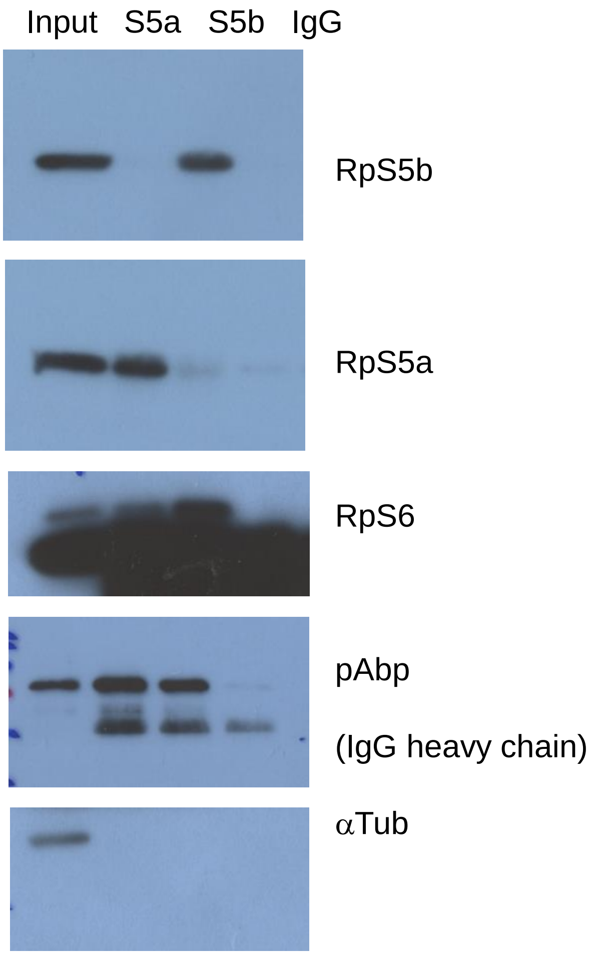

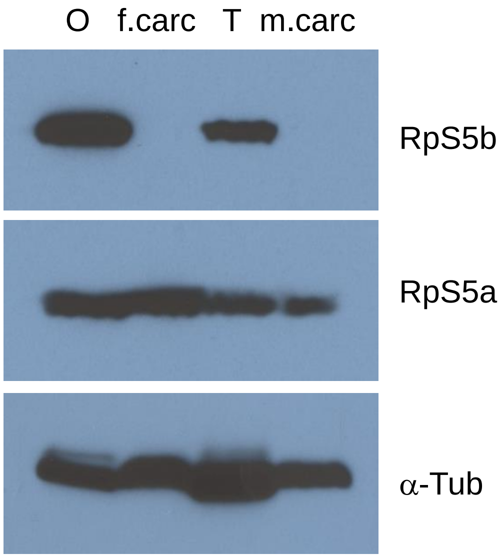

Original scans (ori) for Figure 1B

Figure 1C ori

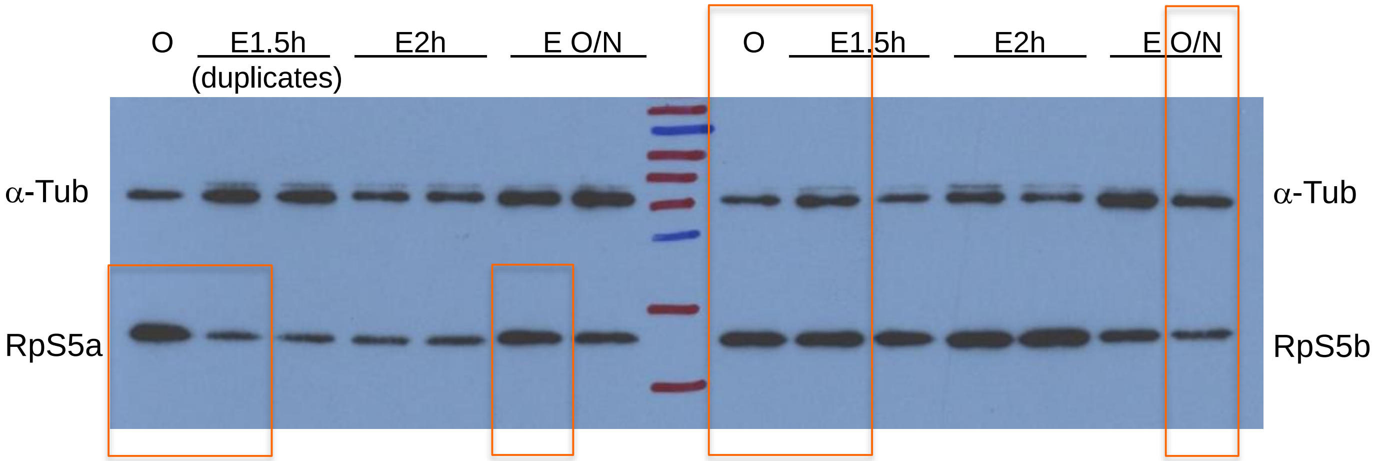

Figure 1D ori

Figure S7 – part 1/3

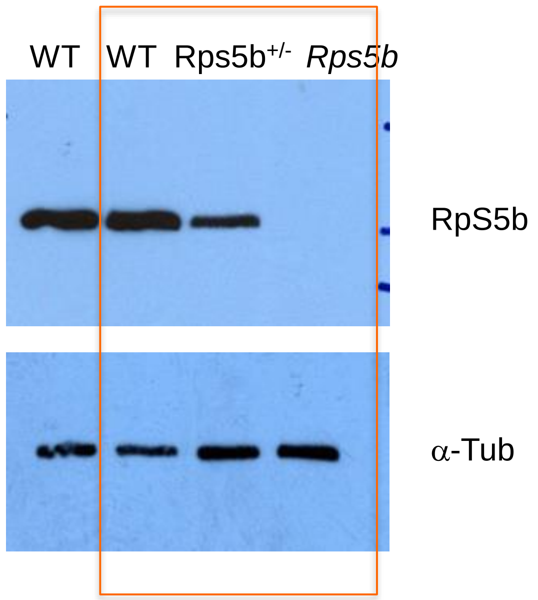

Figure 2A ori

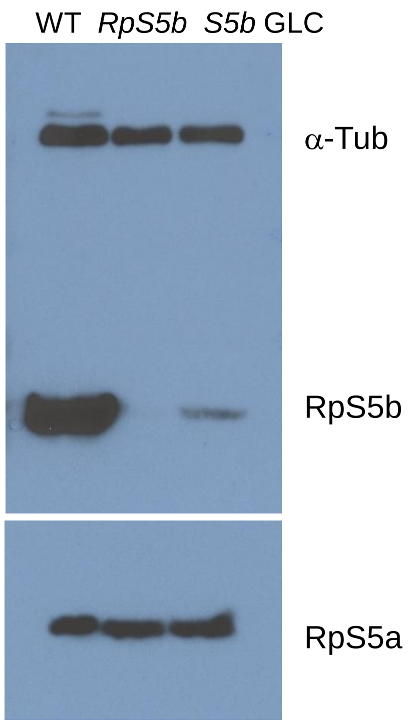

Figure 2Q ori

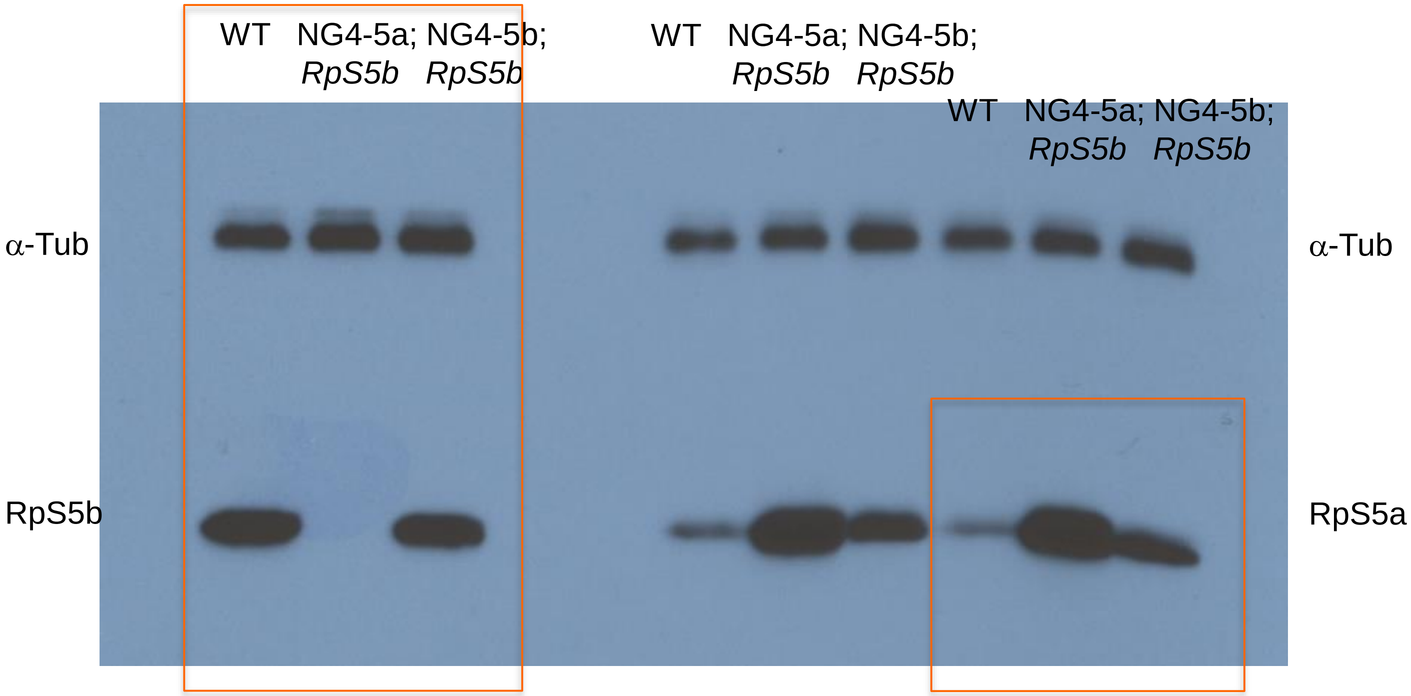

Figure 2X ori

Figure S7 – part 2/3

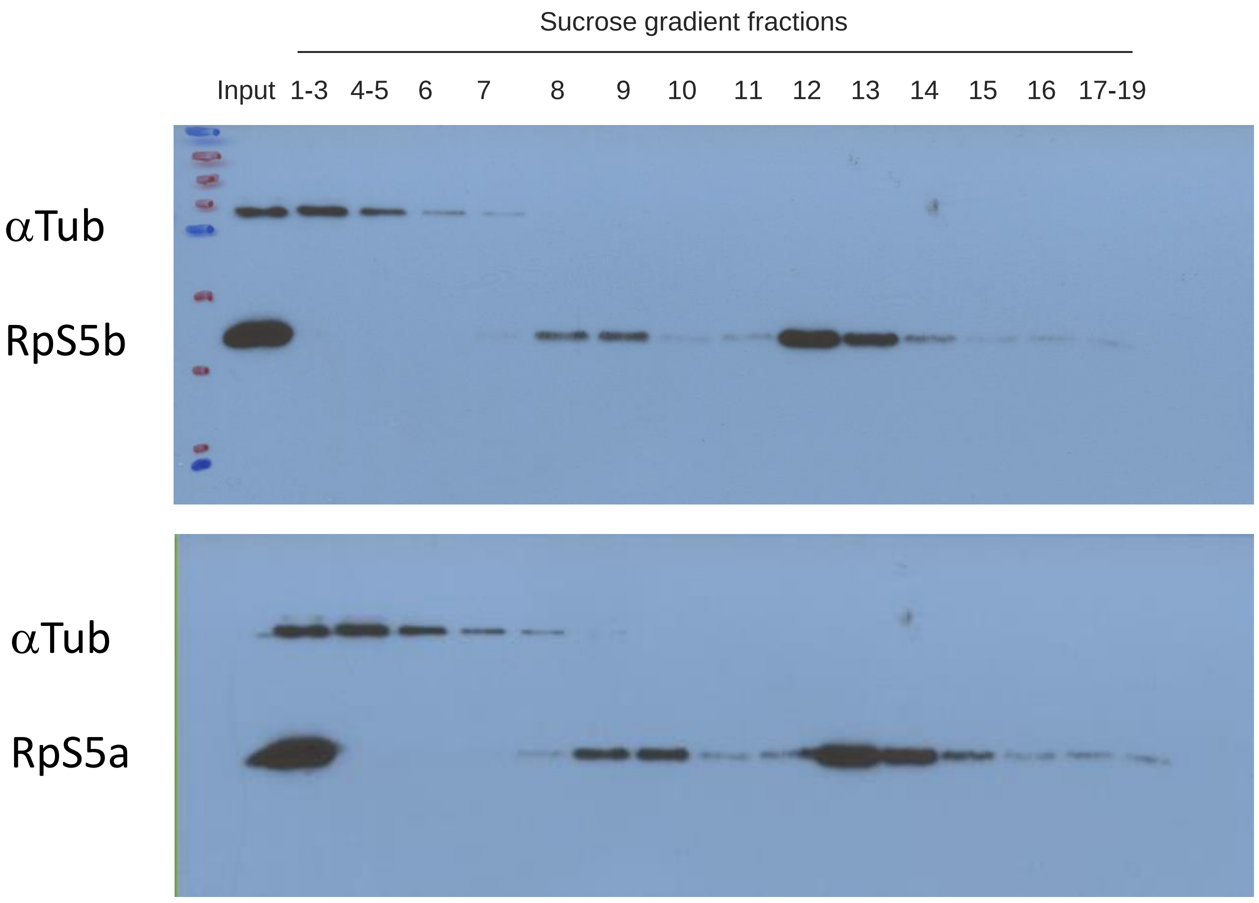

Figure S1 ori

Figure S7 – part 3/3

Supplementary Figure Legends.

Supplementary Fig. S1. (a) RpS5a and RpS5b cosediment with canonical ribosomal protein RpS6 in sucrose gradients. Lysates were prepared from wildtype embryos and fractionated on 15-50% sucrose gradients. Fractions were run on an SDS-PAGE gel, immunoblotted, and incubated with antisera recognizing proteins as indicated. α-Tubulin was used as a control cytosolic protein. **(b)** RpS5a and RpS5b have complementary expression patterns in testes. Immunostaining of whole-mount testes indicates that RpS5a is primarily expressed in somatic cells while RpS5b is primarily expressed in germline.

Supplementary Fig. S2. (a) DAPI staining of *RpS5b* ovaries shows an egg chamber with super numerous nurse cells and two oocytes. **(b, c)** Rhodamine-conjugated phalloidin staining of **(b)** wild-type and **(c)** *RpS5b* ovaries shows excessive accumulation of F-actin in *RpS5b* oocytes. **(d-i')** Transgenic expression of either RpS5 isoform in germline rescues normal morphology. **(d, e)** *nos*-driven expression of untagged RpS5b in germline in the *RpS5b* mutant rescues normal morphology and posterior localization of the oocyte, marked with Orb. **(f, g)** *nos*-driven expression of untagged RpS5a in germline in the *RpS5b* mutant rescues normal morphology and posterior localization of the oocyte, marked with Orb. **(h, h')** *nos*-driven expression of Venus-tagged RpS5b in the *RpS5b* mutant rescues late stages of oogenesis. **(i, i')** *nos*-driven expression of Venus-tagged RpS5a in the *RpS5b* mutant rescues late stages of oogenesis.

Supplementary Fig. S3. Immunostaining experiments with α-Cut (DSHB) on (**a**) wild-type and (**b**) *RpS5b* ovaries showing overproliferation of Cut-positive cells in the mutant.

Supplementary Fig. S4. (a) MA plot of RNA immunoprecipitations from wildtype ovaries with α-RpS5a (RpS5a_IP) or α-RpS5b (RpS5b_IP) compared to input. Statistically enriched (>2 fold, padj < 0.01) and depleted (<2 fold, padj < 0.01) are highlighted in red and blue respectively. M = log2(*pulldown*) - log2(*input*), A = 0.5 * (log2(*pulldown*) + log2(*input*)). Fold changes and adjusted p-values (padj) calculated by DESeq2 (38). The Venn diagram (http://bioinfogp.cnb.csic.es/tools/venny/) shows minimal

overlap between RNAs enriched in populations recruited by RpS5a and RpS5b. **(b)** Heat map representing biological process gene ontology (GO) terms of RNAs enriched in populations recruited by RpS5b and RpS5a, respectively. The most highly significant matches are in red. The fold enrichment of each GO term is plotted in the bar charts. **(c)** Box plot of the length distribution, in nucleotides, of the 5'UTR, coding sequence (CDS) and 3'UTR for RNAs enriched in populations recruited by RpS5a (RpS5a_IP) and RpS5b (RpS5b_IP) (upper panel). Box plots showing the sizes of 5' UTRs, coding sequences (CDS), and 3' UTRs of RNAs associated with RpS5a when RpS5a is expressed under *nos* control (S5a_IP/5a_GL_KD), RNAs associated with RpS5b when RpS5a is expressed under *nos* control (S5b_IP/5a_GL_KD), RNAs associated with RpS5a in the *RpS5b* mutant (S5a_IP/5b-null), as well as RNAs associated with RpS5a and RpS5b in the WT (S5a_IP/WT and S5b_IP/WT, respectively) (lower panel). **(d)** Enriched motifs in RpS5a-associated RNAs (blue) and RpS5b-associated RNAs (orange) as computed by HOMER (37). **(E)** RpS5b-associated RNAs are recruited to RpS5a in the absence of RpS5b. Comparison of mRNA abundance in co-immunoprecipitations from wildtype ovaries with RpS5b (blue), RpS5a (red), or with RpS5a in the *RpS5b* mutant (green). Virtually all these RNAs are more abundant in RpS5a immunoprecipitations from the *RpS5b* mutant than from wildtype.

Supplementary Fig. S5. Heat maps representing the biological process GO terms associated with the proteins **(a)** enriched or **(b)** depleted in the cytosolic fractions from *Rps5b* ovaries as compared with wild-type. The most highly significant matches are in red. The fold enrichment of each GO term is plotted in the bar charts.

Supplementary Fig. S6. *RpS5b* mitochondria cluster more densely and have altered morphology. **(a)** FIB-SEM reconstructions showing mitochondrial distributions in wildtype (WT) and *RpS5b* nurse cells. **(b)** Movie showing z-stack reconstruction illustrating a donut-shaped mitochondrion engulfing other cytosolic material in an *RpS5b* nurse cell.

Supplementary Fig. S7. The original scans for the Western-blots.
